# Non-Linear Diffusion and Power Law Properties of Heterogeneous Systems: Application to Financial Time Series

**DOI:** 10.3390/e20090649

**Published:** 2018-08-30

**Authors:** Miguel A. Fuentes

**Affiliations:** 1 Santa Fe Institute, 1399 Hyde Park Road, Santa Fe, NM 87501, USA; fuentesm@santafe.edu; 2 Instituto de Investigaciones Filosóficas, Bulnes 642, Buenos Aires 1176, Argentina; 3 Facultad de Ingeniería y Tecnología, Universidad San Sebastián, Lota 2465, Santiago 7510157, Chile

**Keywords:** power law, complex systems, non linear evolution equations, 05.70.Ln, 05.30.Pr

## Abstract

In this work, we show that it is possible to obtain important ubiquitous physical characteristics when an aggregation of many systems is taken into account. We discuss the possibility of obtaining not only an anomalous diffusion process, but also a Non-Linear diffusion equation, that leads to a probability distribution, when using a set of non-Markovian processes. This probability distribution shows a power law behavior in the structure of its tails. It also reflects the anomalous transport characteristics of the ensemble of particles. This ubiquitous behavior, with a power law in the diffusive transport and the structure of the probability distribution, is related to a fast fluctuating phenomenon presented in the noise parameter. We discuss all the previous results using a financial time series example.

## 1. Introduction

In recent years, many papers have been written focusing on the study of anomalous collective motions and particularities on probability distributions. In fact, when revising the work done in this area, it is possible to identify different lines of research such as: granular systems [1], turbulence [2], financial processes [3], social dynamics [4,5], among others. We could say that the ubiquitous characteristics that in principle are present in the systems under study have two remarkable properties: power law behavior in the structure of its distribution and dynamic characteristics of a system of many particles with anomalous diffusion (i.e., a power law behavior in its diffusion). The first characteristic occurs in a wide variety of physical, biological and artificial phenomena. Some of these are as dissimilar as the occurrence of the frequency in the use of words, the abundance in the size of biological species, the size of vortices in turbulences, etc. Along with this, it is interesting to note, as we mentioned before, that the characteristics associated with the collective movement of some of these systems, where the second moment, i.e., $<x{\left(t\right)}^{2}>=t^{\alpha }$, being α=1 the value for the exponent where the system behaves in a *normal* way, is the typical quantity to be studied [6].

In this contribution, we will describe particular features at the microscopic level of the system, and how they will impact on the macroscopic characteristics of such behaviors, focusing on a financial time series example. We will first write about the evolution equation of a particle (i.e., a microscopic description), using a Langevin formalism characterized by a stochastic integro–differential equation. Then, we will use a set of similar microscopical systems, to describe the properties of the macroscopical systems (i.e., macroscopic laws). We will arrive to a highly Non-Linear Fokker–Planck equation that was study in relation of nonadditive entropies and complex Systems [7,8]. It has been noticed that the last remark applies in situations where the phase space is partly visited [9]. This approach applies to may situations, as reported elsewhere (see for example [10,11,12] and references therein).

It is important to mention that a central part of our analysis is based on the average of a fluctuating quantity, in an unbalanced equilibrium state of the macroscopical system. The parts that compose this system are cells, or networks of cells, that are characterized to be in a local equilibrium, and can be treated as a collection of similar (or equivalent) particles following the same microscopic dynamics. In that way, we can see the natural emergence of power laws on a compound of mixed complex systems [13].

Also, we will show an application to financial time series. The analysis of these temporal series has a long tradition in statistical physics and complex systems science, see for example [3,14,15,16,17,18,19,20]. In the last section we draw final remarks.

## 2. Microscopic Dynamics

Let us start the presentation by studying the microscopic dynamics of a Brownian particle, where dissipation is described by a memory kernel γ(t). This considers the history of the individual process of the particle (remember that the Brownian particle is influenced by an external noise, which gives unique characteristics to the realization, for each run of the model or trajectory of each particle, for an experiment in real physical systems). The stochastic integro–differential equation reads as follows [21,22]

(1) $$M\ddot{x}\left(t\right)+M\int_{0^{+}}^{t}\gamma \left(t-t^{\prime }\right)\dot{x}\left(t^{\prime }\right)dt^{\prime }+\xi \left(t\right)=0.$$

In the previous equation, ξ(t) characterizes a Gaussian long–range correlated noise. *M* is the mass associated with the particle and γ(t) is a dissipative kernel. We have formally denoted with $0^{+}$ a possible cut–off. For the stochastic term of the equation, we choose ξ(t) as such it has the following properties

(2) $$\begin{array}{ccc} \langle \xi (t)\rangle & = & 0, \end{array}$$

(3) $$\begin{array}{ccc} \langle \xi (t)\xi (0)\rangle & = & 2A_{0}\Gamma \left[\alpha \right]\cos\left(\alpha \pi /2\right)t^{-\alpha }, \end{array}$$

with t>0. The parameter for the coupling strength with the complex bath is $A_{0}$. A microscopic random–matrix model was applied in the study of anomalous diffusions [23,24], used to calculate the kernel γ(t). Then, the (dissipative) kernel γ(t) is defined by

(4) $$Mk_{B}T\;\gamma \left(t\right)=2A_{0}\Gamma \left(\alpha \right)\cos\left(\frac{\alpha \pi }{2}\right)t^{-\alpha }\;,\;t>0.$$

In this equation, α is related with the complexity of the bath. Notice that for non–integer values of α, the bath is called fractal [25]. The behavior of the spectral density is characterized by α when the regime is non-Ohmic [24]. It is also important to mention that if the Riemann–Liouville fractional derivative is introduced

(5) $$\frac{\partial^{r}f\left(t\right)}{\partial t^{r}}=\frac{1}{\Gamma (-r)}\int_{0}^{t}\frac{f(s)ds}{{\left(t-s\right)}^{r+1}},$$

with −1<r<0 [26,27], we can write and equivalente equation for Equation (1) as

(6) $$M\ddot{x}+M\gamma_{\alpha }\frac{\partial^{\alpha -1}\dot{x}}{\partial t^{\alpha -1}}+\xi \left(t\right)=0,$$

which is a fractional Langevin equation. The previous fractional Langevin equation describes the subdiffusion, for 0<α<1, and the superdiffusion regime for 1<α<2. We have also defined $\gamma_{\alpha }$ as follows [28]

(7) $$\gamma_{\alpha }=\frac{\pi A_{0}}{Mk_{B}T\sin\left(\alpha \pi /2\right)}.$$

## 3. Power Law Behavior in the Movement of Ensemble of Particles

We can obtain several dynamical properties from the ensemble of particles, particularly the position of the particle (its distribution) at any time can be calculated via

(8) P(x,t)=∫P(x,V,t)dV,

i.e., the marginal probability distribution. In this equation we observe that $V\left(t\right)\equiv \dot{x}\left(t\right)$. As usual in the case of Gaussian noises the joint probability distribution can be calculated using only a few cumulants [6]. Then, using the second moment

(9) $$\langle x^{2}\left(t\right)\rangle =\frac{2kT}{M}t^{2}E_{2-\alpha ,3}\left(-\gamma_{\alpha }t^{2-\alpha }\right),$$

we can calculate the probability distribution P(x,t). In the previous equation $E_{\mu ,\nu }$ is known as the generalized Mittag–Leffler function [29].

We can observe the second moment in

(10) $$\langle x^{2}\left(t\to \infty \right)\rangle \approx \frac{2kT}{M\gamma_{\alpha }}\frac{t^{\alpha }}{\Gamma (1+\alpha )}\equiv \frac{t^{\alpha }}{b},$$

that shows an anomalous behavior [30], which we explicitly identify with a power law, with $b=M\gamma_{\alpha }\Gamma \left(1+\alpha \right)/\left(2kT\right)$.

From the general previous analysis, we can re-obtain the asymptotic limit for α=1, the classical diffusive transport of the Ornstein–Uhlenbeck process [31]. The evolution for the asymptotic processes corresponds to a diffusion equation, also studied in [32]

(11) $$\frac{\partial P(x,t|b)}{\partial t}=\frac{\alpha t^{\alpha -1}}{2b}\frac{\partial^{2}P\left(x,t|b\right)}{\partial x^{2}}.$$

The last equation can be linked with the fractional Brownian motion (fBm) process [33], see also [34,35,36,37]. This can be done when identifying α=2H with α∈(0,2), so P(x,t|b) is the one time probability distribution of the fBm.

The solution for the last equation in the marginal regime can be written as

(12) $$P\left(x,t|b\right)=\sqrt{\frac{b}{2\pi t^{\alpha }}}\exp\left(-\frac{b\;x^{2}}{2\;t^{\alpha }}\right).$$

## 4. A Marginalization of Weakly Coupled Systems

In the final equation of the last section, we have explicitly noted parameter *b*, i.e., we wrote P(x,t|b). This conditional distribution assigns an event a probability given a particular value of *b*. As the reader can guess, when doing a simple average over a distribution h(b) we can obtain the distribution P(x,t). The resulting distribution, noted here as P(x,t), will be the result of a simple integration

(13) P(x,t)=∫P(x,t|b)h(b)db.

It is worth noting that the distribution h(b) will be determined by the specific spatiotemporal dynamics of the entire system under consideration. For physical systems it is defined on a positive support. One case, among the variety of possible elections, occurs when nearly independent microscopic Gaussian random variables, with average zero, contribute in an additive way to the final dynamics of the system. If *b* is given by the sum

(14) $$b=\sum_{i=1}^{n}x_{i}^{2},$$

then, the distribution of this stochastic variable follows

(15) $$h\left(b\right)=\frac{1}{\Gamma (n/2)}{\left(\frac{n}{2\beta_{0}}\right)}^{n/2}b^{n/2-1}e^{-nb/(2\beta_{0})},$$

which is called Gamma-distribution of order *n*.

Now, if we consider the inverse of *b* (the “temperature” for physical systems), the distribution that naturally arises is the inverse Gamma-distribution

(16) $$h\left(v\right)=\frac{\beta_{0}}{\Gamma (n/2)}{\left(\frac{n\beta_{0}}{2}\right)}^{n/2}v^{n/2-2}e^{-n\beta_{0}/\left(2v\right)}.$$

Also, it is important to mention some important contributions to the field when considering multiplicative noises. Following these lines, if we have a random variable which formally can be expressed by

(17) $$u_{i}=\prod_{i=1}^{n}\xi_{i}$$

where $\xi_{i}$ are *n* random variables, invoking the Central Limit Theorem, we can find that the distribution follows a log-normal distribution

(18) $$h\left(b\right)=\frac{1}{\sqrt{2\pi r}b}e^{-\varphi^{2}}$$

with

(19) $$\varphi =\frac{1}{\sqrt{2}r}\log\left(\frac{h}{m}\right)$$

with *m* and $r^{2}$ as mean and variance.

These types of distributions give rise to the distribution P(x,t) with a slow decay, sometimes more complex than the simple power law behavior [38].

Important analytical results can be seen if we perform the marginalization over Equation (9) using the distribution written in Equation (13). We can find the evolution equation for the complete system as

(20) $$\frac{\partial P(x,t)}{\partial t}=D\left(t\right)\frac{\partial^{2}P{\left(x,t\right)}^{\frac{n-1}{n+1}}}{\partial x^{2}},$$

where the diffusion parameter D(t) follows

(21) $$D\left(t\right)\propto t^{\frac{n\alpha }{n-1}-\frac{n+1}{n-1}}.$$

The distribution that satisfies this equation presents a clear power law behavior

(22) $$P\left(x,t\right)=\sqrt{\frac{\beta_{0}}{\pi nt^{\alpha }}}\;\frac{\Gamma \left[\frac{\left(n+1\right)}{2}\right]}{\Gamma \left[\frac{n}{2}\right]}{\left(\frac{\beta_{0}}{n}\frac{x^{2}}{t^{\alpha }}+1\right)}^{-\frac{n+1}{2}},$$

as can be seen, this characteristic is more critical for larger values of *x*.

## 5. Application to the Financial Time Series

In this section, we will discuss an application of the previously discussed theoretical approach and results. We will show how we can understand the anomalous diffusion and the characteristics of the distribution of returns (the logarithm of the fraction of the prices) when considering those from the beginning of the process. With this definition of return we can appreciate that, as a new results, the process shows not only a fat tail in its distribution, but also a clear anomalous diffusion process.

We will use the time series generated by the New York Stock Exchange (NYSE) during one year for a highly traded stock: the International Business Machines (IBM).

As usual we will define the return as the difference of the price logarithm, but now taking into account the beginning of the daily series

(23) $$r\left(t\right)=\ln\left[p\left(t\right)\right]-\ln\left[p\left(t_{0}\right)\right].$$

In this definition, p(t) is the price defined as the midpoint between the best bid and offer price in the market (this is known as “quotes”). There are several ways to set the unit of the time index, time *t*. Here time is updated whenever an event causes change in the midpoint between the prevailing best quotes (this is the finest possible time scale).

Following Reference [39], we computed $<r{\left(t\right)}^{2}>$ measuring each day the second moment at different times.

**Figure 1:**
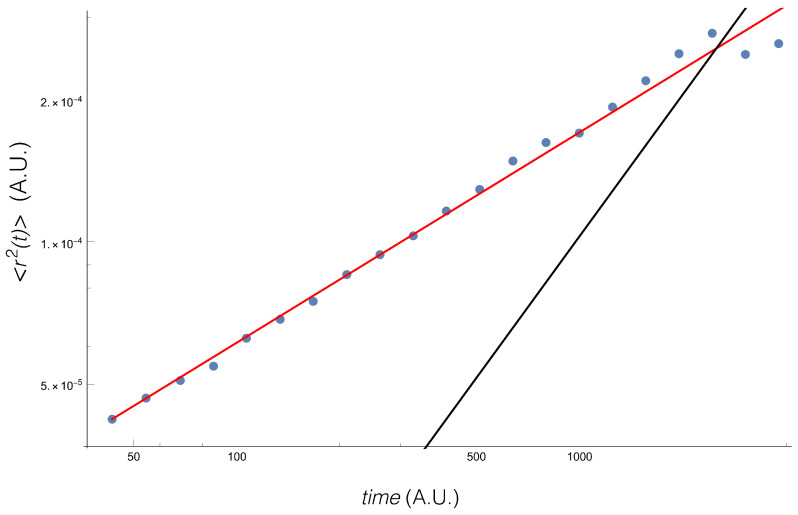
Second moment of the return.The red line corresponds to the empirical fit α=0.44, while the black line shows α=1.

The result (Figure 1) shows the well known fact that the diffusion is anomalous, with an exponent α=0.44.

We then measured the different values of *b*, the inverse of the variance, for each day. We obtained the gamma distribution written in Equation (15) and performed the integration, Equation (13). Following these steps we found the analytic distribution Equation (22).

**Figure 2:**
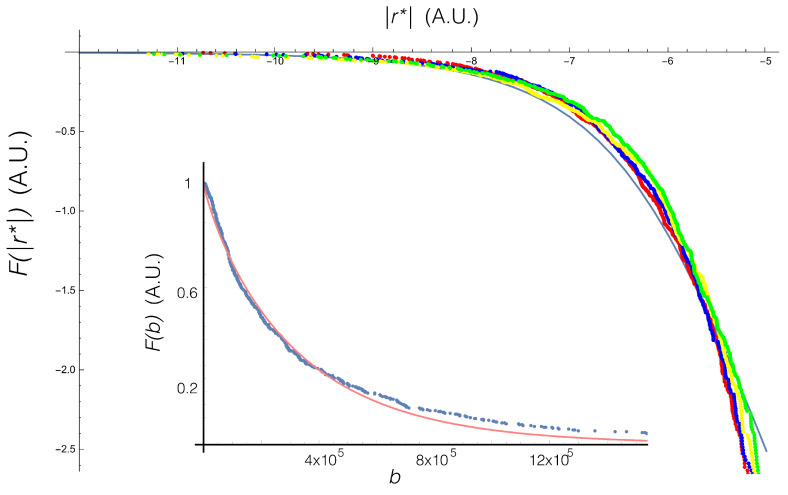
Collapse of the empirical complementarycumulative distribution for time t=100, 200, 1000 and 2000. The continuous blue line is the theoretical curve, after the marginalization Equation (13). Inset: Complementary cumulative distribution for *b*, and the fit to a gamma distribution.

In Figure 2 we show the collapse of the complementary cumulative distribution *F*, where is defined (for a given f(x)) as

(24) $$F=1-\int_{0}^{\infty }f\left(x\right)dx$$

and

(25) $$r*=\frac{r}{t^{\alpha /2}}.$$

The figure shows the empirical result for various times (different colors in the figure). The analytical result is also shown. We include an inset showing the complementary cumulative distribution for the parameter *b*.

## 6. Final Remarks

In the present work, we have shown that it is possible to obtain important behaviors, ubiquitous in many systems. First, we found that power laws are not only present in the distribution of variables that are relevant in the understanding of a physical problem, but also in the dynamical properties of them. We have also shown an analytical way to connect the microscopic characteristics of a single particle, with the microscopic characteristics of the full system. Among the main results obtained we find that anomalous behaviors appear in the financial time series when considering the price at time zero as a reference for the return. Another result is the possibility of obtaining the fat tail distribution when using the same approach. It is worth noting that the previous results also holds (for α=1) for a temporally homogeneous Gauss–Markov process, like the mentioned Ornstein–Uhlenbeck process. In this case the function to be marginalized is the ubiquitous Gaussian distribution.
